# Evolution of sexually dimorphic longevity in humans

**DOI:** 10.18632/aging.100640

**Published:** 2014-02-22

**Authors:** David Gems

**Affiliations:** Institute of Healthy Ageing, and Department of Genetics, Evolution and Environment, University College London, London WC1E 6BT, UK

**Keywords:** aging, eunuch, evolution, gender gap, menopause, polygyny, testosterone

## Abstract

Why do humans live longer than other higher primates? Why do women live longer than men? What is the significance of the menopause? Answers to these questions may be sought by reference to the mechanisms by which human aging might have evolved. Here, an evolutionary hypothesis is presented that could answer all three questions, based on the following suppositions. First, that the evolution of increased human longevity was driven by increased late-life reproduction by men in polygynous primordial societies. Second, that the lack of a corresponding increase in female reproductive lifespan reflects evolutionary constraint on late-life oocyte production. Third, that antagonistic pleiotropy acting on androgen-generated secondary sexual characteristics in men increased reproductive success earlier in life, but shortened lifespan. That the gender gap in aging is attributable to androgens appears more likely given a recent report of exceptional longevity in eunuchs. Yet androgen depletion therapy, now used to treat prostatic hyperplasia, appears to accelerate other aspects of aging (e.g. cardiovascular disease). One possibility is that low levels of androgens throughout life reduces aging rate, but late-life androgen depletion does not.

The pattern of human aging exhibits a number of salient features that have long engaged evolutionary biologists. For one, among the higher primates, human being are unusually long lived. The maximum lifespans of orangutans and gorillas are 58.7 and 54 years, respectively, and those of our closest relatives, bonobos and chimpanzees are 50 and 53.4 years, respectively [1]. By contrast, maximum human lifespan varies from 85 in foraging groups such as the Aché in Paraguay and Kung bushmen, to 122 in the developed world [1, 2]. This implies that an evolutionary spurt of increased longevity must have occurred since the last common ancestor of humans and chimpanzees/bonobos walked the earth some 5-7 million years ago [1].

Another striking feature of human aging is its sexual inequality. As life expectancy has increased with improvements in living conditions during the last century, there has consistently emerged a survival difference between the sexes, with women living longer [3, 4]. For example, in the UK estimated life expectancies for women and men from birth (2012) are 82.4 and 78.0 years, respectively, a difference of 4.4 years [5]. Some other examples of gender gaps are the USA 5.0 years (81.0F - 76.0M), France 6.4 years (84.7F - 78.3M) and Russia 13.0 years (73.1F - 60.1M). The gender gap reflects a greater susceptibility of men to a wide range of aging related pathologies, including cardiovascular disease, type II diabetes, infection and sarcopenia (aging-related loss of muscle mass) [3]. The basis of male frailty remains unclear, either in terms of its evolutionary origins or somatic causes.

The other gender gap in aging affects reproductive lifespan. Women's capacity reproduce is lost in their late 40s, as they undergo the menopause, while men can remain fertile at least into their early 80s. The significance of the early cessation of reproduction in women is a topic of much discussion, in particular, whether or not it is an adaptation and contributes to evolutionary fitness.

Aging itself does not appear to be an evolutionary adaptation. Instead, it occurs because the effects of genes on viability at advanced ages make little contribution to fitness. This is because in the wild, few individuals will survive to those ages due to external causes of mortality (e.g. starvation, predation, disease) [6]. For example, a mutation with deleterious effects on health in mice that is expressed only after 3 years of age will experience no selection against it, since in the wild mice almost never survive that long. Thus, the evolved lifespan of a species broadly reflects the intensity of extrinsic mortality in its usual niche, and its capacity to reproduce later in life.

A given gene can exert multiple effects on phenotype at different points in the life history. Such pleiotropy means that a given gene may, in principle, exert beneficial effects early in life, but deleterious ones later in life (antagonistic pleiotropy). Because early life traits are more important to fitness, such genes can accumulate in populations, leading to aging [7].

The evolutionary theory of aging suggests a number of hypotheses about how the distinctive pattern of human aging might have come into existence. Human longevity must have evolved as the result of reduced extrinsic mortality, which allowed increased reproduction in late life. The emergence of human intelligence and language use likely helped to reduce extrinsic causes of death such as predation. Evolved sexual dimorphism in aging can result from sex differences in extrinsic mortality and age-specific reproductive output [7]. For example, our male ancestors might have experienced greater extrinsic mortality relative to our female ancestors due, say, to mate seeking and hunting. Regarding the menopause, the possibility that it is an adaptation has been extensively considered [7-9]. The Grandmother Theory proposes that post-menopausal women increase the reproductive success of their younger relatives [10-12]. In this way, the genetic determinants for the menopause are maintained in populations because of their effects on inclusive fitness, i.e. women with menopause can produce more offspring equivalents in later life by caring for the children of relatives than through their own pregnancies.

Previous reviews have surveyed the various ideas proposed about the significance of the menopause, e.g. [7-9], and about the cause of the gender gap in human aging, e.g. [4, 13-15]. This essay proposes a synthesis of several earlier ideas about human evolution with observations on the longevity of eunuchs, thereby offering a plausible account of how we may have come to age as we do.

## Human longevity and late-life reproduction in men: the patriarch hypothesis

Increases in lifespan evolve because they support increased reproduction in later life. In the case of humans, increased longevity supports increases in late-life reproduction in men but not in women (at least, not directly). In fact, the reproductive lifespan of women is little different to that of other higher primates: the age at last birth in female chimpanzees and women is 42 and 45, years respectively [1]. This suggests that human longevity evolved as the result of selection for late-life reproduction in the males of our hominin ancestors [16, 17].

This deduction is central to the patriarch hypothesis [18], which postulates that increased intelligence and the possibility of accumulating resources allowed successful older males to continue to reproduce in later life by acquiring additional, younger wives. Existence of such long-lasting polygynous families (i.e. polygamy with one husband and several wives) would be supported by accumulation of resources. In fact, our species may well have evolved from polygynous ancestors. Although monogamy is currently widespread, in traditional societies polygyny is widespread, if not the predominant arrangement. Moreover, polygyny among our ancestors is supported by Y chromosome diversity analysis [19].

**Figure 1:**
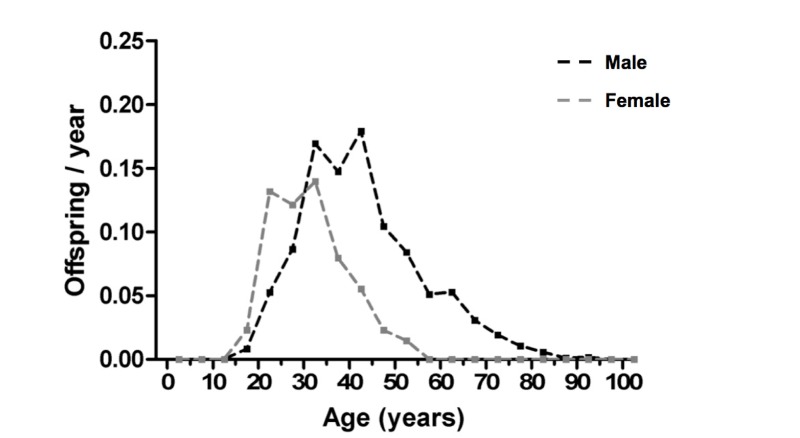
Polygyny and sexual dimorphism in reproductive output This shows an example of fertility distributions in a polygynous population (Northern Ghana) [17, 20] (figure kindly prepared by D. van Bodegom).

Patterns of age-specific reproduction in men and women in polygynous societies typically show marked sex differences. For example, in the Bimoba and Kusasi peoples of Northern Ghana, female reproductive output peaks by 20 yrs of age, and then declines from the mid 30s, with very few offspring after 50; by contrast, male reproductive output increases from the late teens until around 30 yrs of age (Figure 1) [17, 20]. Age increases in reproductive output are predicted to create a strong selection for increased longevity [7]. Moreover, male reproductive output continues up to the 9th decade, clearly reflecting conception with younger wives.

Marlowe and others suggest that the lack of an increase in reproductive lifespan in women reflects evolutionary constraint, stemming from sex differences in the mechanisms by which gametes are produced [18, 21]. While in men new sperm are produced by the testes throughout adulthood (iterogametogenesis) [22], women produce their entire stock of oocytes during embryogenesis. As these age, they undergo programmed cell death at an ever increasing rate, probably to assure successful reproduction by precluding production of defective embryos. Rates of oocyte loss appear much the same in female chimpanzees as in humans [23]. Thus, to evolve a longer reproductive female lifespan would require a radical redesign of the primate ovary. This view implies that the menopause, like aging itself, is non-adaptive, and replaces the Grandmother theory with a more bleak perspective. This is perhaps consistent with a historically recurrent trend of mistreatment of older women as a group, for example witch-burning in medieval Europe [24] and, as noted by Darwin, the cannibalistic consumption of older women by Fuegian indians during times of food scarcity [25]. However, if evolutionary constraint were the cause of the menopause, this does not exclude a contribution of post-menopausal women to inclusive fitness. For example, in a recent study of a polygynous communities in Ghana, the presence of post-menopausal women was associated with a 2.7% increase in the number of babies born to younger women [17].

The possibility that human longevity evolved as the result of reproduction by older men in polygynous family groups has some fascinating implications. Polygamy linked to reproductive longevity, involving colonies with a single reproductive individual, is a recurrent pattern in the animal kingdom. For example, in eusocial insects such as ants, where queens can attain great longevity (e.g. up to 28 years in the black garden ant *Lasius niger*) [26]. Thus, the aging reproductive male sheltered within his large family group may experience reduced extrinsic mortality and increased late-life reproduction, much as does the ant queen in her nest [27]. This family structure may have played a critical role in the evolution of human longevity. A further implication is that the switch from polygyny to monogamy has removed the major selective force for the evolution of human longevity.

It also raises the possibility that ancestral hominin females might have increased their reproductive success by behaving in a way that would maximise the probability that their male offspring would have a long reproductive lifespan. One means to secure this goal would be selection of males with a long reproductive lifespan, i.e. exerting female choice to mate selectively with older males. Moreover, selection of older males is a means by which females can ensure lower frequency of mutation in her offspring, since males with fewer mutations will better survive to older ages [28]. Female choice leading to mating with older males has been observed in diverse organisms including fish (e.g. redlip blennies) [29], birds (e.g. Tengmalm's owl) [30] and non-primate mammals (e.g. African elephants) [31].

An intriguing (if speculative) possibility is that sexual selection for greater age occurred among our hominin and human ancestors, favouring males who appeared older than they actually were. This might explain the enigma of male pattern baldness, which is a secondary sexual characteristic under the control of testicular hormones [32, 33]. It is notable that even today, women still tend to marry men older than themselves. For example, according to U.S. census data for 1999, 33% of women married men >4 years older than themselves, while only 6.4% married men >4 years younger, a 5.2-fold difference (couples, *N* = 55,849) [34].

## Why are men the shorter-lived sex? Eunuchs and antagonistic pleiotropy

According to the patriarch hypothesis, the longevity of women is the result of the inheritance of autosomal genetic determinants of longevity selected for in men. But this raises the question [17]: if longevity evolved in men, why then are women longer lived than men? Here a further sex difference in human reproductive patterns provides a plausible explanation: the greater variance in male reproductive output. In women, the number of offspring is limited by the number of successful pregnancies, while in men it is limited by the number of obtainable mates. Moreover, polygyny implies that some males sire no progeny [17]. This creates a strong selective pressure in favour of traits that promote male reproductive success, e.g. increased musculature [14]. At least some such traits will appear among secondary sexual characteristics, under the control of male-specific endocrine factors, including androgens such as testosterone and its more bioactive derivative dihydrotestosterone (DHT).

Such strong selection for male reproductive success may well have led to the evolution of trade offs between early life benefits in terms of establishment as a patriarch, and later costs in terms of the detrimental effects on late-life health of the male endocrine patterns [18]. By this view, the contemporary pattern of male aging is the result of antagonistic, selective forces. First, later life selection due to the patriarch's protected position, status and access to younger wives extends lifespan. Second, early life selection on traits that improve the probability of becoming a patriarch in the first place create an endocrine status that eventually has pathogenic effects, shortening lifespan, an instance of antagonistic pleiotropy [14, 35].

This scenario predicts that removal of the testes (castration) might extend male lifespan. This possibility is supported by a study of mentally disabled men who had the misfortune to live in the USA in the early-mid 20^th^ century, where sterilization of the “genetically unfit” was common as a result of policies initiated by the eugenics movement [36]. Analysis of mortality data from 297 castrated men, and 735 age-matched intact controls revealed a significant increase in lifespan in the former (70.7 vs. 64.7 yrs, p < 0.001) [37]. If only those castrated earlier in life were considered, the effect on lifespan was more profound: an increase in median lifespan of 11.6 years.

Although the Hamilton and Mestler study supports the idea of a life-shortening effect of possession of testes, it remained possible that such benefits of castration are peculiar to institutionalized mentally handicapped males. Notably, the life expectancy of intact control males was considerably lower than that of the general population [37]. Moreover, there is no clear relationship between testosterone levels and lifespan in intact men in retrospective studies; one of US veterans over the age of 40 found an association between low testosterone and *increased* all-cause mortality, even after adjustment e.g. for age, medical morbidity and BMI [38].

This issue has been explored further through studies of eunuchs (castrated men) who historically were a significant presence in a number of societies, from Yugoslavia to China [39, 40]. Notable eunuchs in Western Europe included the castrato singers, who underwent prepubertal castration to retain in adulthood their prepubescent vocal range (e.g. soprano and mezzo-soprano). One study compared the lifespans of 50 Italian castratos with a set of intact male singers of the same period, and detected only a 1.2 year increase in mean lifespan in the castratos, that was not statistically significant (65.5±13.8 yrs *vs.* 64.3±14.1 yrs) [41]. However, the lack of statistical significant here could reflect the small sample size [42]. A further possibility is that some would-be castrates were in fact intact men whose voices had not broken at puberty.

This long-standing controversy appears to have been resolved by publication in 2012 of a study of eunuchs at the Imperial Court of the Chosun Dynasty in Korea [43]. There, eunuchs could attain high official ranks, and genealogical records of eunuchs exist that include birth and death dates. From such records of the period 1556-1861, 81 eunuchs were identified, with a mean lifespan of 70.0±1.76 years (range 27-109). This compared to mean lifespans of intact men of comparable social status ranging from 50.9-55.6 years, i.e. eunuchs lived 14.4-19.1 years longer than intact men. Moreover, three of the 81 eunuchs became centenarians, living to 100, 101 and 109 years, a frequency that is at least 130-times higher than in contemporary developed societies [43]. This new study confirms the conclusion of Hamilton and Mestler (1969), that the presence of testes markedly shortens lifespan. It is also consistent with the idea that the testes are a determinant of the gender gap in human lifespan.

## Modern eunuchs: androgen deprivation therapy and late-life health

Discovering the biological basis of the gender gap could allow the development of interventions that provide additional years of healthy life to men. Of course, most men would not consider orchiectomy (castration), particularly given the consequent loss of masculine attributes, including genital hypotrophy, loss of libido, gynecomastia and development of a more feminine body fat distribution pattern [40]. But the recent report on Korean eunuchs will provide food for thought to male-to-female transsexuals, some of who undergo orchiectomy. (Effects of gender reassignment on aging remains unclear, since this was only performed in large numbers from the 1970s; however, recent findings suggest no change in mortality rates [44]).

A likely mechanism by which testes exert effects on late-life health is through production of testosterone, whose secretion by the Leydig cells is stimulated by pituitary luteinizing hormone (LH). Testosterone is converted to its more potent derivative dihydrotesto-sterone (DHT) by 5α-reductase (5αR) enzymes, e.g. by type II 5αR in the prostate gland. These androgens certainly seem to have detrimental effects on health, e.g. by suppressing immunity [45] and promoting unhealthy lipoprotein profiles [46, 47]. Androgens have an especially bad effect on the prostate gland, promoting benign prostatic hyperplasia (BPH) and, less frequently, prostate cancer [35, 48]. By the age of 80 at least 75% of men develop BPH, which largely affects glandular and stromal tissue of the transition zone of the prostate [49].

Development of BPH and prostate cancer is dependent upon the presence of the testes, and orchiectomy before the age of 40 prevents it [44, 50]. Promotion of BPH by the testes is mediated at least in part by testicular androgens, since blocking conversion of testosterone to DHT by drugs such as the 5α-reductase inhibitor finasteride inhibits BPH [48]. However, other testicular factors probably also play a role [35].

The use of 5α-reductase inhibitors for androgen-deprivation therapy (ADT) is now widely applied to treat advanced prostate cancer [48]. Effectively, ADT is a form of chemical castration, and its widespread use has created a new population of modern eunuchs. Whether ADT slows male aging has been a topic of speculation [35] which the new study of Korean eunuchs underscores. Is it likely that, like the Korean eunuchs, contemporary men undergoing ADT will live longer? BPH aside, is it likely to protect them against the rest of the aging process?

## Androgens and cardiovascular disease

One reason for the gender gap is that men are more prone to cardiovascular disease [51], and though it has been suggested that androgens contribute to this [52], much evidence argues against this [51]. For example, a recent meta-analysis of 70 studies showed an association between low testosterone levels and increased risk of cardiovascular disease [53]. This is particularly seen in cross-sectional studies, i.e. comparing their health and androgen levels among older men. Here a confounding factor is that ill health can markedly depress androgen levels [38, 53]. Thus, more informative is data from long-term effects of ADT. However, here again, an increase in CVD incidence is seen, e.g. [54, 55], reviewed in [53]. Moreover, a meta-analysis of 30 placebo-controlled tests of effects of testosterone replacement therapy (TRT) detected no increase in risk of CVD [56]. Consistent with this, CVD was not reduced among 297 eunuchs in the U.S. [37], but was among 989 institutionalized eunuchs in Denmark [57]. ADT can also increase insulin resistance, hyperinsulemia and hyperglycemia [58].

Thus, testosterone promotes aging in at least one organ (the prostate) but not in several others (e.g. the cardiovascular system, the musculature), but there is evidence that removal of testes can extend overall lifespan, at least in some settings (e.g. the Korean Imperial Court).

How to reconcile these findings? One possibility is that testicular factors other than androgens play a role in the control of aging. Another is that androgen depletion from childhood or early adulthood is beneficial, but androgen depletion initiated in later life is largely detrimental. Here it is notable that for Chosun court eunuchs, castration was typically performed early in life, on boys (i.e. before puberty) [43]. This is consistent with the greater increases in lifespan seen by Hamilton and Mestler when castration was performed earlier in life [37]. These observations support the view that it is during puberty and early adulthood that testicular androgens induce developmental changes that promote pathology in later life [15].

## Prostate hyperplasia as a model for the evolution of aging

The evolution of late-life BPH is likely explained by the principle of antagonistic pleiotropy, where increased androgen levels and/or altered prostatic response to it increased early life fitness but in later life gave rise to hyperplasia [7, 35, 59]. Thus, BPH is a good model for investigating the proximate biological mechanisms of the evolution of aging. Here a key question is: what are the selective advantages conferred by the alterations in prostate function that eventually lead to BPH?

A major function of the prostate is semen production: in humans it makes 50–75% of seminal volume. This raises the possibility that selective advantage in sperm competition is important here. Given that sperm competition should not be an important issue in polygynous families, this suggests that BPH may be an ancestral trait that evolved before the appearance of polygyny. Consistent with this, one of the only other mammalian species known to develop BPH is our close relative, the chimpanzee [60]. Notably, the relatively large size of human testes relative to body size suggests a degree of ancestral promiscuity, as seen in chimps, rather than exclusive polygyny, as seen in gorillas, which have smaller testes [61].

Antagonistic selection affecting mechanisms of sperm competition and lifespan have been studied in detail in the fruitfly *Drosophila melanogaster* [62]. Here, complex effects are exerted by peptides in the seminal fluid, secreted by the accessory glands, equivalent in function to the mammalian prostate gland [63]. For example, sex peptides in fly seminal fluid increase female egg production but decrease female lifespan [64]. Similarly, prostatic fluid contains hundreds of proteins, including immunosuppressants (e.g. prostaglandins, TGFβ1) and antibacterial peptides. For example, TGFβ1 acts to protect sperm within the female by suppressing female host immunity, but in later life in the male causes fibroblast to myofibroblast transdifferentiation within the prostate, leading to epithelial cell hypertrophy [49]. This illustrates how, at the molecular and cellular levels, androgens may orchestrate trade-offs between early life sex-related fitness traits and late-life pathologies.

## Conclusions

**Figure 2:**
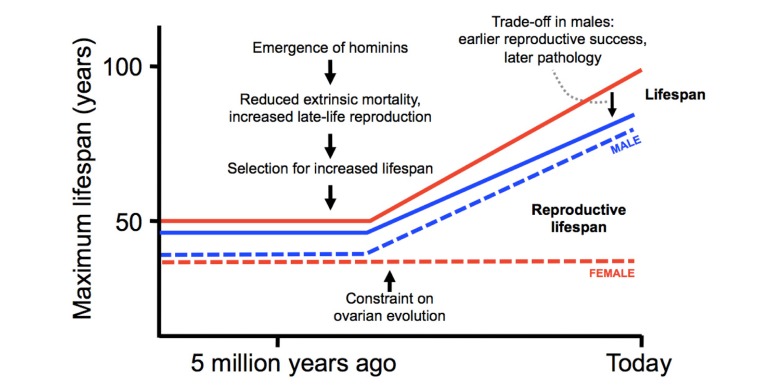
Evolution of modern pattern of human aging Hypothetical scheme showing the evolutionary origins of human longevity (relative to other higher primates), the gender gap in lifespan, and the menopause. Numbers and curves are intended to represent general trends rather than precise values.

According to the account presented here, summarized in Figure 2, the current pattern of human aging is the result of three key factors. Increased late-life reproduction by men has increased human longevity relative to other higher primates (the patriarch hypothesis) [18]; the design of the ovary has constrained the evolution of longer reproductive lifespan in women [65]; and antagonistic pleiotropy acting on testicular function has decreased male lifespan [35].

The study in 2012 by Min et al. demonstrating marked longevity in eunuchs of the Korean Imperial Court [43] provides new support for the idea that testicular function increases late-life pathology and mortality in men. One possibility is that castration unmasks underlying male constitutional longevity [66], i.e. the effects of autosomal determinants of human longevity that resulted from selection in males, but which are normally more strongly expressed in females [17]. Perhaps consistent with this, human males show delayed maturity relative to females [67]. Yet although androgens do promote some aspects of age-related pathology (e.g. prostate cancer), they appear to protect against many others (e.g. cardiovascular disease, sarcopenia, osteoporosis) [53]. One possibility is that it is during puberty and early adulthood that androgens exert an effect that decreases late life health. What would be particularly helpful at this stage is additional studies of aging in eunuchs.
